# Decoupling of Interfacial Ionic Diffusion from Segmental Dynamics in Silica-Filled Silicone Gel through Experiments

**DOI:** 10.1021/acs.langmuir.5c01650

**Published:** 2025-06-17

**Authors:** Ying Lin, Chuanle Heng, Yuhao Liu, Yifan Xu, Tao Wen, Antonio Facchetti, Lijian Ding

**Affiliations:** † School of Electrical and Automation Engineering, 558979 Hefei University of Technology, Hefei 230009, China; ‡ College of Electrical Engineering and Automation, 12423 Fuzhou University, Fuzhou 350108, China; § School of Materials Science and Engineering, Georgia Institute of Technology, Atlanta 30332, Georgia, United States

## Abstract

This study experimentally investigates and models the ionic diffusion coefficient at inert particle/polymer interfaces in silica-filled silicone gels, aiming to elucidate the relationship between ion diffusion and segmental dynamics at interfaces. As the specific surface area of silica increases, more loop-type structure chains form at the silica–silicone gel interface. Enhanced silicone chain flexibility at the interface, coupled with a low dielectric constant, strengthens electrostatic interactions and elastic forces, increasing ion jump energy barriers and reducing ion diffusion. Consequently, the influence of polymer segmental dynamics on ion diffusion becomes more significant, shifting the decoupling index between segmental dynamics and the ionic diffusion coefficient from negative to zero. This study provides a theoretical understanding of how interfacial chain movement impacts ion diffusion at interfaces supported by experiments, which is critical to modeling electrical properties of composite polymers and optimizing the performance of relevant electronic and future bioelectronic devices.

## Graphical abstract

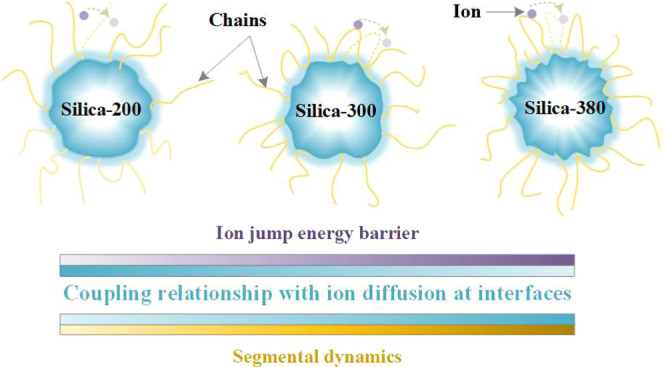

## Introduction

Inert particles are often added to polymers to promote mechanical strengths, electrical properties, and thermal conduction abilities for applications in electrical devices, transportation, construction, etc.^1–3^ For inert particle-filled polymers, such as polymer electrolytes or insulating polymers, the ionic conductivity is a key parameter determining whether the composites can be utilized.^4^ Therefore, understanding the ionic conductivity in polymer composites is vital for manipulating their properties and extending their applications in various fields such as well-established electrical energy storage,^5^ energy conversion,^6^ and insulation equipment,^7^ as well as in new areas such as bioelectronics where solid electrolytes are needed.^8,9^

Ionic charge transport in polymeric materials has been extensively studied. In classical theories,^6,10,11^ below the glass transition temperature (*T*_g_), the ions jump from one site to another over an energy barrier that is controlled by electrostatic interactions and elastic forces according to the Arrhenius equation. Above the *T*_g_, ion diffusion is assisted by the movement of molecular segments, following the Vogel–Tammann–Fulcher (VTF) equation. Recent studies revealed that even above the *T*_g_, there also exist decoupling relationships between the polymer chain relaxation and ion diffusion, that is, ions could move through the free volume between the rigid chains.^12,13^

Based on the deep understanding of ion transport in polymers, extensive efforts have been directed to explore the effect of particles on the ion diffusion in polymer composites after it was reported that the addition of inert particles enhanced the ionic conductivity of polymers, such as poly­(ethylene oxide) (PEO) and poly­(ethylene glycol)­(PEG) by several orders of magnitude.^14,15^ It was found that inert fillers could improve the ionic conductivity of composites via the formation of a highly conducting inert particle/polymer interface region which increases the free volume at the interface.^16^ This occurs by changing the chain segmental dynamics at the interface,^17^ via Lewis acid–base interactions between the filler and the polymer,^15^ forming a space-charge region at the interface.^11^ This data indicates that the inert particles change the physical and chemical states of polymers in the vicinity of particles to influence the interfacial ion diffusion process and eventually act on the ion diffusion behavior and ionic conductivity of whole composites. However, most studies always investigate the ionic conductivity of the whole composites to infer how the particles alter the ion diffusion at the interface instead of characterizing interfacial ion transport directly.^16,18–23^

Recently, the effect of chain conformation and dynamics of the polymer segment on ion mobility at interfaces by molecular dynamics simulations has been shown.^24–26^ Nevertheless, capturing the ion diffusion the particle/polymer interface by experiments remains absent as also stated by Guo et al.^11^ Furthermore, similar to the ion diffusion in polymers above the *T*_g_, the ion diffusion at interfaces should also be affected by the chain dynamics at interfaces since the interfacial region has polymer chains. Although the broadband dielectric spectrum method can detect and distinguish molecular chain dynamics in different amorphous phases, the interfacial segments’ movement strength is always weak. The abundant dielectric responses may cover the interfacial segments’ relaxation, which also increases the difficulty in understanding interfacial segments’ dynamics. Therefore, it remains unclear how the segment dynamics acts on the ion diffusion at interfaces.

In this paper, we propose a calculation method for the ionic diffusion coefficient at particle/polymer interfaces based on the effective medium theory. Furthermore, using a silica-filled silicone gel composite as a testbed, which is a typical inert particle-filled polymer widely applied in electronic device encapsulation, sealing materials, and insulator fields,^27–29^ the decoupling between the ionic diffusion coefficient and the segmental relaxation at interfaces is investigated experimentally. Our findings highlight the relationship between ion diffusion and segmental dynamics at inert particle/polymer interfaces, enhancing the understanding and providing guidance for the manipulation of ionic conductivities in composite polymer materials.

### Calculation Method for Segmental Relaxations and Ionic Diffusion Coefficients at Particle/Polymer Interfaces

The key parameters in segmental dynamics and ionic diffusion are the molecular chain relaxation time and ionic diffusion coefficients, respectively. It has been widely reported that the broadband dielectric spectrum (BDS) is sensitive to detect the interfacial polymer chain relaxation α_int_, which follows the Vogel–Tamman–Fulcher (VTF) equation as^30^

1 $$\tau_{\mathrm{int}}=\tau_{0}\;\exp\left(\frac{BT_{0}}{T-T_{0}}\right)$$

where τ_int_ is the relaxation time of relaxation α_int_ observed in the BDS, τ_0_ is the constant relaxation time, *T*_0_ is the Vogel temperature, *B* is related to the fragile strength, and *T* is the absolute temperature. Definitions of all symbols can be found in Table S1 in the Supporting Information.

However, it is challenging to determine the ionic diffusion coefficients at the interface since the scale of particle/polymer interfaces is too small to measure, and molecular simulations always model the local region instead of all interface information inside a composite. The diffusion coefficients can be traced back to the basic dc conduction equation and the Nernst–Einstein equation as^31^

2 $$D_{\mathrm{int}}=\frac{\sigma_{\mathrm{int}}k_{B}T}{q^{2}n_{0}}$$

where *D*_int_ is the ionic diffusion coefficient at the interface, σ_int_ is the ionic conductivity at the interface, *k*_B_ is the Boltzmann’s constant, *q* is the charge of ions, and *n*_0_ is the concentration of ions. If the parameters *q, n*_0_, and σ_int_ are known, then the ionic diffusion coefficient at interfaces can be obtained.

Note, *q* ^2^ *n*_0_ can be calculated as

3 $$q^{2}n_{0}=\frac{\sigma_{\mathrm{cp}}k_{B}T}{D_{\mathrm{cp}}}$$

where *D*_cp_ and σ_cp_ are the ionic diffusion coefficient and conductivity of the composites, respectively. The σ_cp_ is easy to measure or fit by BDS data. Based on the Trukhan model,^12^ the *D*_cp_ can be also calculated from the electrode polarization effect in the BDS as *D*_cp_ = 2π*f*_max_ *L* ^2^/[32­(tan θ)_max_^2^].

Here, (tanθ)_max_ is the maximum value of dielectric loss in the frequency range of electrode polarization, *f*_max_ is the frequency corresponding to (tanθ)_max_, and *L* is the thickness of tested composites.

For calculating the ion conductivity at the particle/polymer interface, we employed the effective medium theory proposed by Nan and Smith.^32^ Therefore, if the total conductivity is known, the ionic conductivity at the interface can be deduced. In the theory, the matrix is an ionic conductor, the particles are insulators, and a highly conductive interface layer coats the particles,^32^ which is similar to our system.^33^ Hence, based on the effective medium theory, the method to calculate the ion conductivity at particle/polymer interfaces is proposed.

The first step is to calculate the volume fraction of particles in a composite unit *Y* (the silica particle along with its interfacial area), as shown in Figure 1, which can lead to different interface ionic conductivity equations in the effective medium theory.^32^ *Y* = 1/(1+*d*/*R*)^3^, where *R* is the radius of the particles, and *d* is the thickness of the interface layer. Our previous work demonstrated how to calculate the thickness of an interface layer based on BDS data.^34^ The volume fraction of fillers *V*_filler_ in the whole sample is *V*_filler_ = (*m*_filler_/ρ_filler_)/(*m*_filler_/ρ_filler_+ *m*_matrix_/ρ_matrix_), where *m*_filler_ and ρ_filler_ are the weight and density of fillers, and *m*_matrix_ and ρ_matrix_ are the weight and density of matrixes. If the relaxation strength of polymer chains in matrixes Δ*ε*_matrix_ representing the content of the matrix and the relaxation strength of interfacial polymer chains Δ*ε*_int_ representing the content of the interface can be found in the BDS data,^35^ the volume fraction of interfaces *V*_int_ in the whole sample can be deduced from *V*_filler_ + *V*_int_(1 + Δ*ε*_matrix_/Δ*ε*_int_) = 1. Therefore, *d* obeys (4π­(*d* + *R*)^3^/3 – 4π*R* ^3^/3)/(4π*R* ^3^/3)= *V*_int_/*V*_filler_. *Y* can be calculated as

4 $$Y=\frac{\left(1+\Delta \varepsilon_{\mathrm{matrix}}/\Delta \varepsilon_{\mathrm{int}}\right)V_{\mathrm{filler}}}{1-V_{\mathrm{filler}}\Delta \varepsilon_{\mathrm{matrix}}/\Delta \varepsilon_{\mathrm{int}}}$$

**Figure 1:**
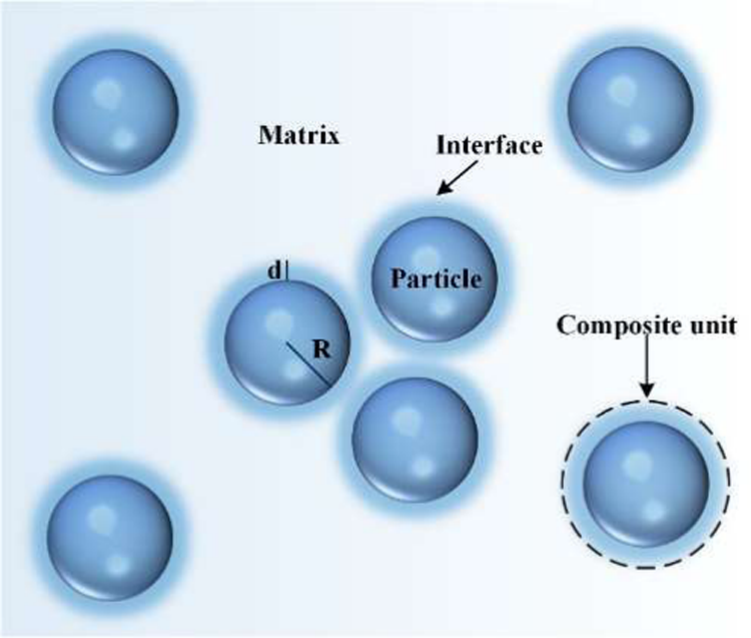
Particle-filled polymer matrix scheme in effective medium theory.

The second step is to compare *V*_filler_ with *Y*. If *V*_filler_ is less than *Y*, according to the effective medium theory,

5 $$\sigma_{0}^{a}=\sigma_{0}\frac{2Y-2V_{filler}}{2Y+V_{filler}}$$

6 $$\frac{V_{filler}}{Y}\frac{\sigma_{c}^{a}-\sigma_{cp}}{\sigma_{cp}+p_{c}\left(\sigma_{c}^{a}-\sigma_{cp}\right)}+\left(1-\frac{V_{filler}}{Y}\right)\frac{\sigma_{0}^{a}-\sigma_{cp}}{\sigma_{cp}+p_{c}\left(\sigma_{0}^{a}-\sigma_{cp}\right)}=0$$

7 $$\sigma_{c}^{a}=\sigma_{c}\frac{2V_{\mathrm{filler}}}{3Y-V_{\mathrm{filler}}}$$

8 $$\sigma_{c}=\sigma_{\mathrm{int}}\frac{2\sigma_{\mathrm{int}}+\sigma_{\mathrm{filler}}+2Y\left(\sigma_{\mathrm{filler}}-\sigma_{\mathrm{int}}\right)}{2\sigma_{\mathrm{int}}+\sigma_{\mathrm{filler}}-Y\left(\sigma_{\mathrm{filler}}-\sigma_{\mathrm{int}}\right)}$$

where σ_c_^a^ and σ_0_^a^ are modified conductivity parameters. σ_0_ is the conductivity of the matrix, which can be tested or fitted from the BDS data of the polymer. *p*_c_, the continuous percolation threshold for the fillers, can be set to 0.28 on the basis of general percolation theory.^32^ From eqs 5 and 6, σ_c_^a^ can be calculated. Based on eq 7, the equivalent conductivity of the composite unit σ_c_ is obtained. According to the σ_c_ and conductivity of the fillers σ_filler_, σ_int_ is eventually found by eq 8. On the other condition that *V*_filler_ is not less than *Y*, in the effective medium theory,

9 $$\sigma_{2}^{b}=\sigma_{\mathrm{filler}}\frac{2V_{\mathrm{filler}}-2Y}{3-V_{\mathrm{filler}}+Y}$$

10 $$\left(1-V_{\mathrm{filler}}\right)\frac{\sigma_{c}^{b}-\sigma_{\mathrm{cp}}}{\sigma_{\mathrm{cp}}+P_{c}\left(\sigma_{c}^{b}-\sigma_{\mathrm{cp}}\right)}+\left(V_{\mathrm{filler}}-Y\right)\frac{\sigma_{2}^{b}-\sigma_{\mathrm{cp}}}{\sigma_{\mathrm{cp}}+P_{c}\left(\sigma_{2}^{b}-\sigma_{\mathrm{cp}}\right)}=0$$

11 $$\sigma_{c}^{b}=\sigma_{c}\frac{2-2V_{\mathrm{filler}}+2Y}{2+V_{\mathrm{filler}}-Y}$$

where σ_c_^b^ and σ_2_^b^ are modified conductivity parameters. *P*_c_ can be taken as the percolation threshold of a general random mixture, i.e., 0.15. Similarly, by combining eqs 9, 10, 11, and 8, the ion conductivity at interfaces can be calculated.

After obtaining σ_int_ and the parameters *q* and *n* based on eq 2, the ionic diffusion coefficient at the interface can be obtained. Subsequently, comparing the temperature-dependent relaxation time of interfacial polymer chains τ_int_ and temperature-dependent ionic diffusion coefficients at interfaces *D*_int_, based on Angell decoupling theory and Sokolov’s reports,^12,36^ the decoupling relationship between both should be revealed theoretically as

12 $$D_{\mathrm{int}}\propto \tau_{\mathrm{int}}^{n-1}$$

The decoupling exponent *n* corresponds to the degree of decoupling between the molecular chain movement and ion diffusion process at the interface. If *n* = 0, it indicates that ion transport is totally dominated by polymer chain relaxation. If the absolute value of *n* is far away from 0, it means that there are other main factors influencing ion diffusion.

## Experimental Section

### Materials and Preparation of Silica-Filled Silicone Gel Composites

Part A (90 g) and part B (9 g) of the silicone gel SEMICOSIL 915 HT (Wacker Chemical Co., Ltd., Germen) were mixed in a beaker by a mixer (Shanghai Lichen Instrument Technology Co., Ltd.) at a rotation rate of 200 rpm for 5 min, which is marked as sample S000 (density 0.97 g/cm^3^). When the silica addition amount exceeds 2.7 g, the viscosity of silicone gel composites is too high and the fluidity is too low to generate the smooth surface of cured samples. Therefore, silica (2.7 g) with a specific surface area of BET 200, 300, and 380 m^2^/g (density 2.3 lb/cu·ft, Shanghai Macklin Biochemical Co., Ltd., China) was added into the mixed silicone gel (99 g) in beakers and mixed uniformly by the same mixer at a rotation rate of 200 rpm for 15 min, which are labeled as samples S200, S300, and S380, respectively. Afterward, the uncured silicone gel S000, S200, S300, and S380 were poured into disk Teflon molds with a diameter of 40 mm and a thickness of 2 mm. The molds were put in vacuum for 1 h to avoid the bubbles inside the sample. Finally, the molds were set in the oven at 100 °C for 1 h to cure the silicone gel.

### Characterization

The upper and lower surfaces of the disc samples S000, S200, S300, and S380 were sputtered with gold by an ion sputtering apparatus (SD-900M, Beijing Boyuan Micro Nano Technology Co., Ltd.). The BDS of the cured sample S000, S200, S300, and S380 were measured at a frequency of 10^–2^–10^4^ Hz from 50 to 110 °C at an interval of 10 °C under a voltage of 3.0 V by a BDS machine (Novocontrol Concept 80, German) to illuminate the electrode polarization effect, matrix polymer chain relaxation, and dc conduction. The BDS of the samples were also tested at a frequency of 5 × 10^1^–5 × 10^4^ Hz from −30 to −60 °C at an interval of 5 °C under a voltage of 3.0 V to elucidate the interfacial polymer chain relaxation.

A scanning electron microscope (SEM) Gemini 500 (Carl Zeiss AG, Germany) was used to observe the dispersion and measure the size of the silica in the composites after samples were teared, and the cross-sections were sprayed with gold. For each figure of samples S200, S300, and S380, three points where the silica fillers are exposed mostly on the cross-section of composites are picked and the diameters of the points are measured.

## Results and Discussion

### Broadband Dielectric Spectrum of Silica-Filled Silicone Gel Samples

The measured BDS data of silica-filled silicone gel are shown in Figures 2 and[Fig fig3]. In Figure 2a–d, two clear steps can be found in the real part of complex permittivity, which refer to the EP relaxation caused by electrode polarization and relaxation α_v_ caused by the movement of polymer chains in matrixes. Meanwhile, in the imaginary part of complex permittivity, a large slope line concealing the relaxation EP and relaxation α_v_ can be observed, which refers to the dc conduction. As shown in Figure 3a–d, there is no relaxation in pure silicone gel shown in Figure 3a2, while relaxations α_int_ are all observed in filled silicone gel, which corresponds to the polymer chain movement at silica/silicone gel interfaces. More details about how to distinguish the relaxations and dc conductions in silica-filled silicone gel can be found in our previous work.^33^

**Figure 2:**
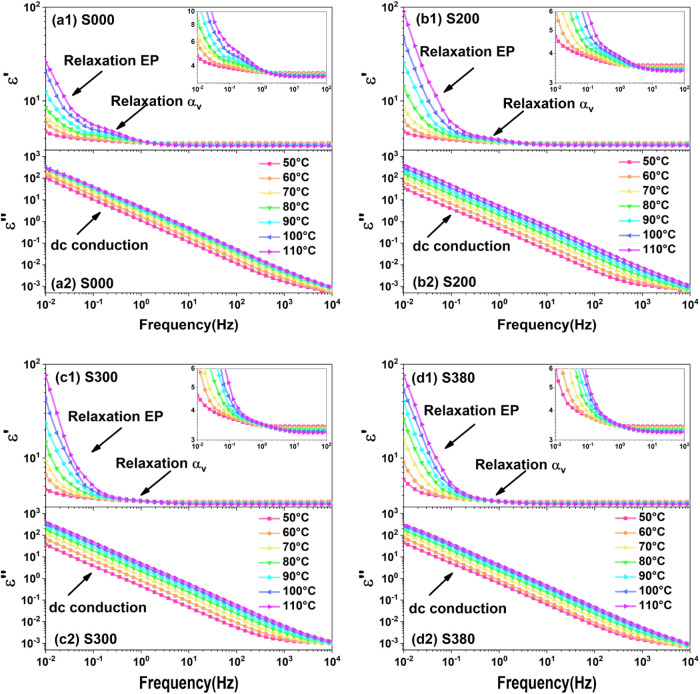
Broadband dielectric spectra of silicone gel samples at high temperatures. (a1–d1) Real part of complex permittivity of samples S000, S200, S300, and S380, in which the relaxation α_v_ step is locally magnified. (a2–d2) Imaginary part of complex permittivity at high temperatures.

**Figure 3:**
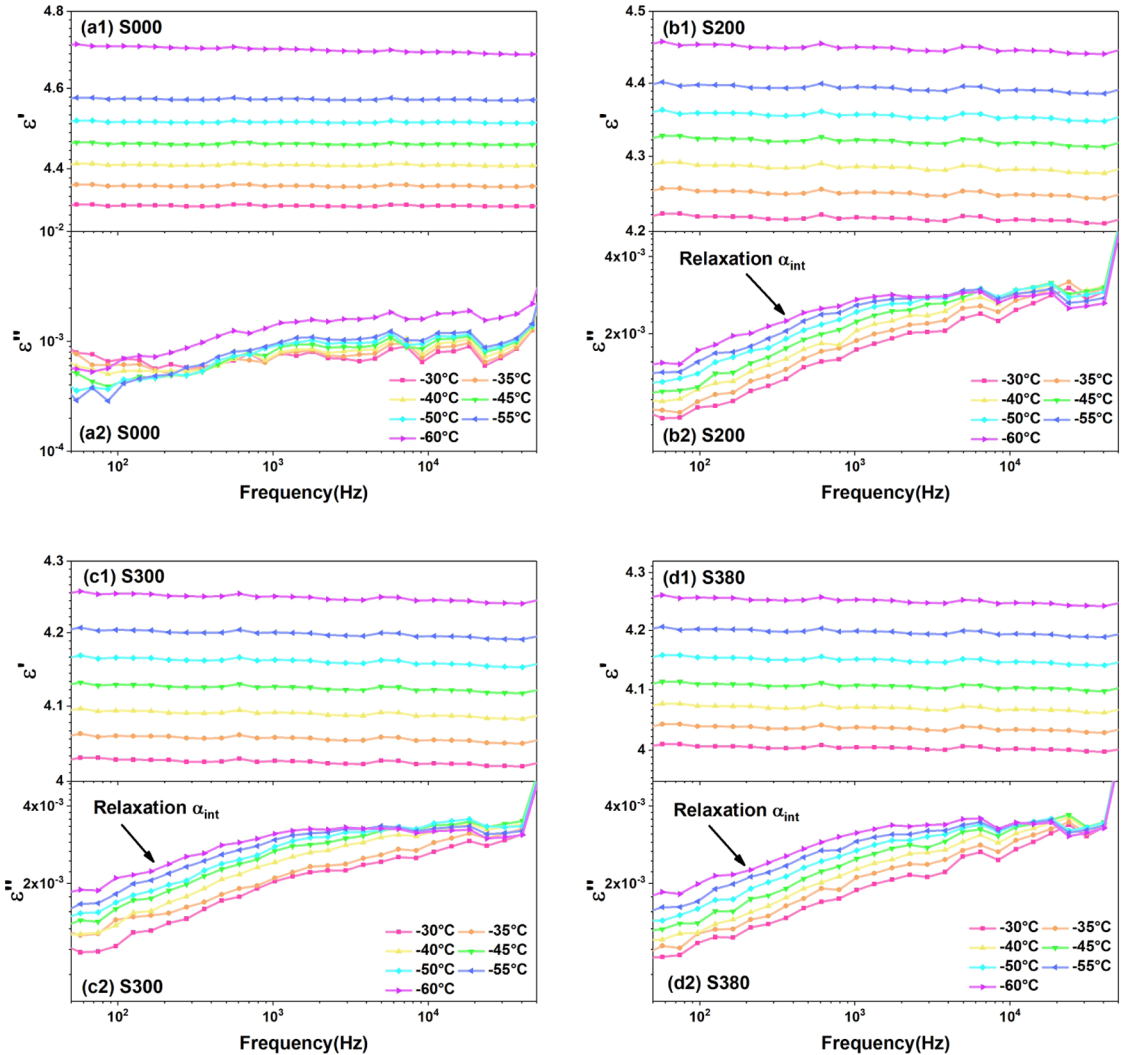
Broadband dielectric spectra of silicone gel samples at low temperatures. (a1–d1) Real part of complex permittivity of samples S000, S200, S300, and S380. (a2–d2) Imaginary part of complex permittivity at low temperatures.

To obtain accurate dc conduction and relaxation information, quantitative fitting of the BDS data is necessary. Eq 13 describing the dc conduction and Cole–Cole model is adopted as^37,38^

13 $$\varepsilon^{*}=\varepsilon_{\infty }+\sum_{i=1}^{s}\frac{\Delta \varepsilon_{i}}{1+{\left(j\omega \tau_{i}\right)}^{\alpha_{i}}}-j\frac{\sigma_{\mathrm{dc}}}{\varepsilon_{0}\omega }$$

where ε* is the complex permittivity; *ε*_∞_ is the permittivity at high frequency; *s* is the number of relaxation peaks; Δ*ε*_i_ is the dielectric strength of *i*th relaxation; τ_i_ is the relaxation time of *i*th relaxation; α_i_ is the parameter depending on the shape of *i*th relaxation (0< α_i_ < 1); *j* ^2^ = −1; ω is the angular frequency; *ε*_0_ is the vacuum permittivity; and σ_dc_ is the dc conductivity. As shown in Figure 2a–d, there are two relaxations (*s* = 2), dc conduction, and permittivity at high frequency. As shown in Figure 3a–d, the dc conduction is very small and can be neglected. The permittivity at high frequency and the relaxation α_int_ contribute to complex permittivity.

The fitting is conducted by the Levenberg–Marquardt method and the universal global optimization method in firstopt software, in which the real and imaginary parts are fitted at the same time. The data of sample S200 at 110 °C and sample S380 at −60 °C are utilized as examples to demonstrate the accuracy of fitting, as shown in Figure 4. The squares of correlation coefficients of both are 0.99987 and 0.99998, respectively, which indicates an excellent fitting effect.

**Figure 4:**
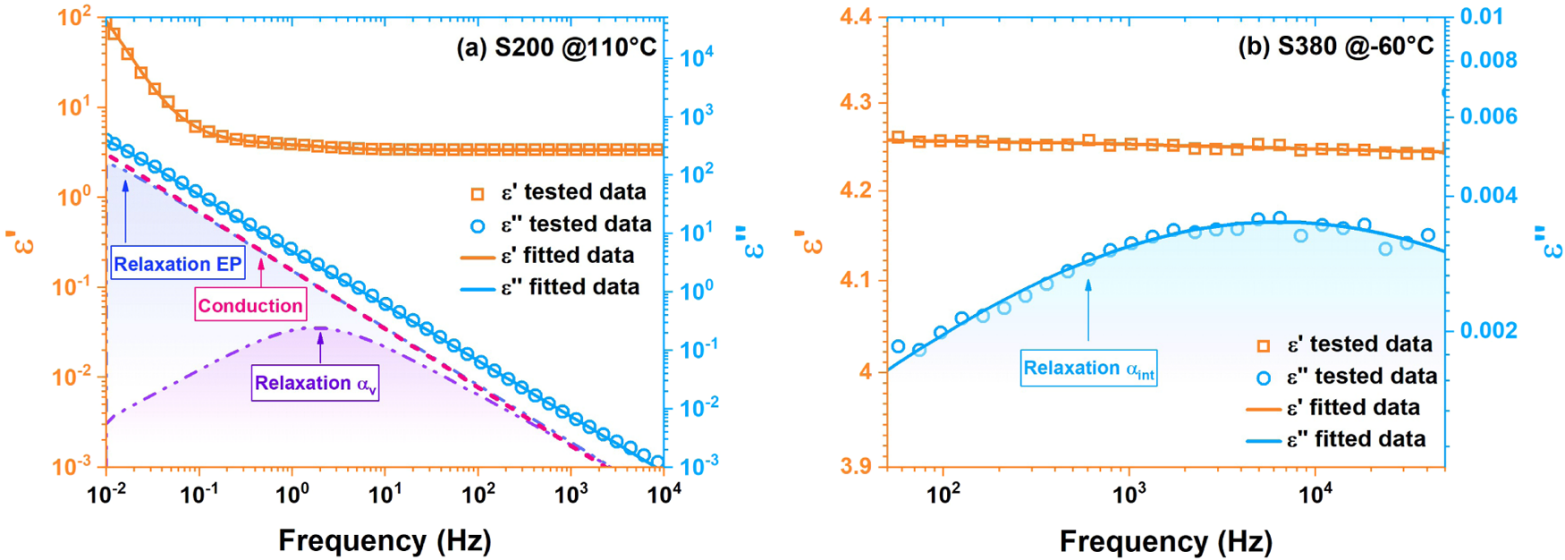
Fitting examples of BDS data. (a) Data of sample S200 at 110 °C and (b) data of sample S380 at −60 °C.

### Temperature-Dependent Segmental Relaxation at Silica/Silicone Gel Interfaces

After extracting the important data in the BDS, the segmental dynamics at the silica/silicone gel interfaces is analyzed in this section. The extracted interfacial polymer chain relaxation time τ_int_ from Figure 3b–d also obeys the VTF equation in eq 1, where τ_0_ is set as 10^–13^ s,^39^ as shown in Figure 5a. The squares of correlation coefficients are 0.9996, 0.9956, and 0.9983 for samples S200, S300, and S380, which displays an excellent fitting. The cooperative rearrangement ability of interfacial polymer chains *m*_int_ can be estimated by^40^

14 $$m_{\mathrm{int}}=16+590/B$$

**Figure 5:**
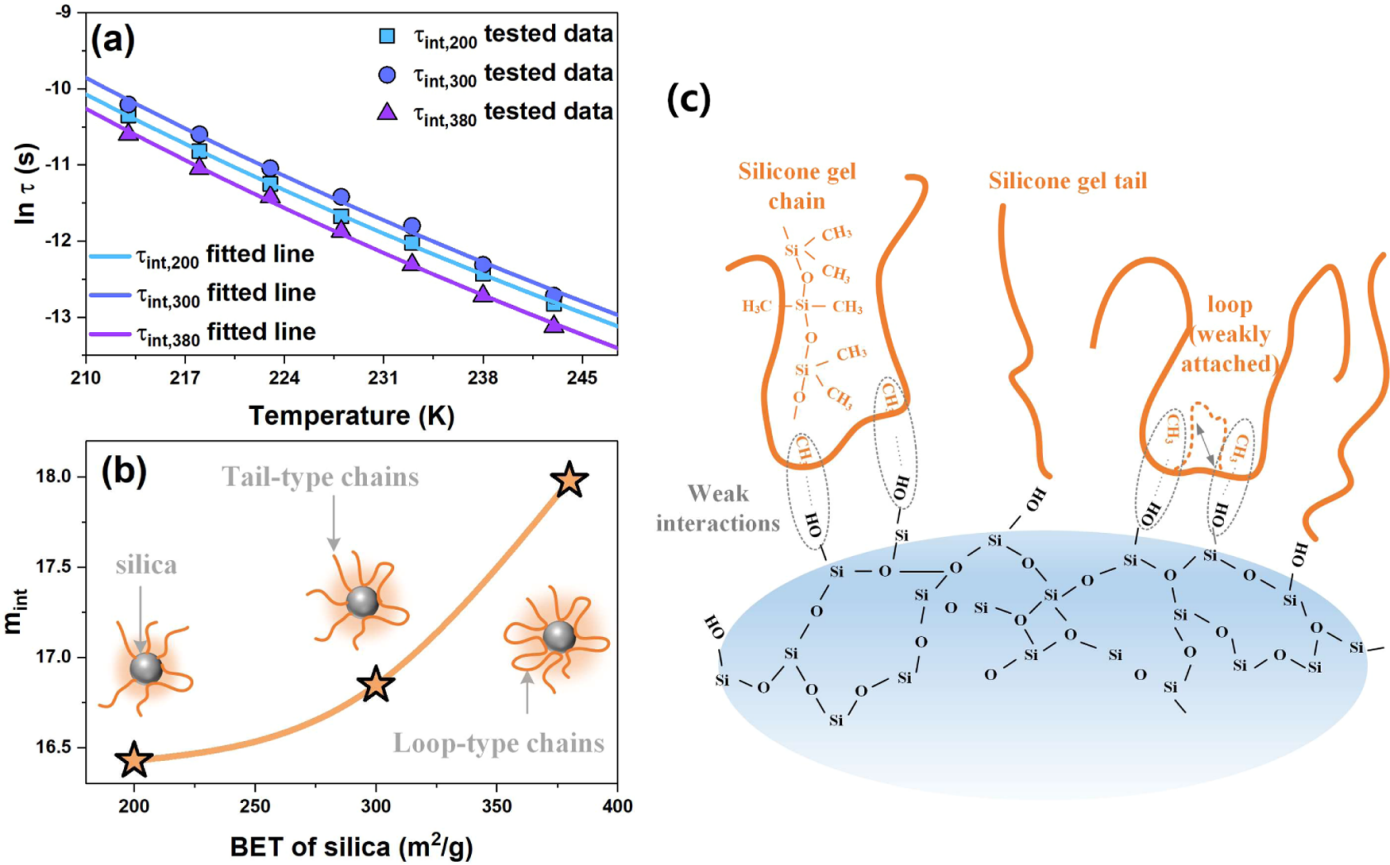
(a) Temperature-dependent relaxation times of interfacial polymer chains, with the relaxation times τ_int,200_, τ_int,300_, and τ_int,380_ of the polymer chains at 200, 300, and 380 m^2^/g silica/silicone gel interfaces. (b) Cooperative rearrangement ability of interfacial polymer chains in composites with different BET silica, where *m*_int,200_, *m*_int,300_, and *m*_int,380_ correspond to the cooperative rearrangement ability of the polymer chains at 200, 300, and 380 m^2^/g silica/silicone gel interfaces. (c) Schematic of the chemical structures at silica/silicone gel interfaces.

Higher *m*_int_ values indicate interfacial polymer chains with greater flexibility and ability to cooperatively rearrange. Upon increasing the specific surface area of silica, *m*_int_ increases, as shown in Figure 5b. This phenomenon has been reported for the silica-filled polydimethylsiloxane materials.^41^ A higher specific surface area increases the contact points of the silicone gel chains with the silica surface. This promotes the formation of more loop-type structures and fewer tail-type structures at the silica/silicone gel interface, as illustrated in Figure 5b,c. The corresponding functional groups of silicone gel and silica are shown in Figure S1 in the SI and the silica/silicone gel interfacial adhesion effect is great, which is also calculated, as shown in Figure S2 in the SI. The high mobility and cooperativity of the loop-type structures enhance the flexibility of the silica/silicone gel interface, thereby facilitating segmental movement at the interface.

### Temperature-Dependent Ionic Diffusion Coefficient at the Silica/Silicone Gel Interface

After the interface segmental dynamics is understood, ionic diffusion coefficients can be quantified. Based on the fitting results of relaxation EP and dc conductivities of the composites, *D*_cp_, *D*_0_, and *q* ^2^ *n*_0_ are calculated by eq 3, as shown in Figure 6 and Table S2. Next, the volume fraction of particles in the composite unit *Y* is calculated. The SEM images for samples S200, S300, and S380 are shown in Figure 7. The average radii of silica particles are 7.71, 7.32, and 7.78 μm for samples S200, S300, and S380, respectively, which means that the particle size is similar and our composites are consistent with the system shown in Figure 1. The relaxation strength of interfacial polymer chains remains stable with the temperature, as shown in Figure 3b–d. Therefore, it is inferred that due to the strong interactions of silica with the polymer at the interface, the relaxation strength of interfacial polymer chains changes little with the temperature.^34^ According to the weight composition and the density of the composites, *V*_filler_ can be calculated, and fitting the BDS data with eq 4 affords Δ*ε*_matrix_ and Δ*ε*_int_, *Y*, and the results including interfacial thickness are summarized in Table S2.

**Figure 6:**
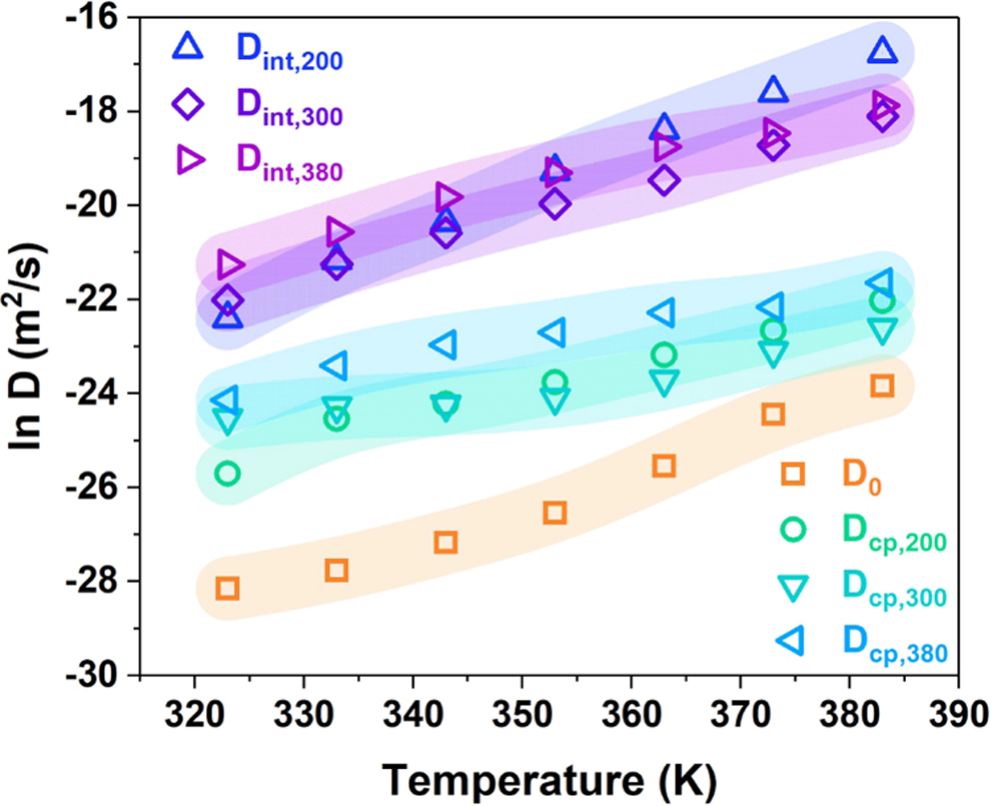
Ionic diffusion coefficients of pure silicone gel *D*_0_, silica-filled silicone gel composites *D*_cp,200_, *D*_cp,300_, and *D*_cp,380_, and silica/silicone gel interfaces *D*_int,200_, *D*_int,300_, and *D*_int,380_, where subscripts 200, 300, and 380 refer to the specific surface area of silica particles. The color bands are connected lines among the data to depict the trend.

**Figure 7:**
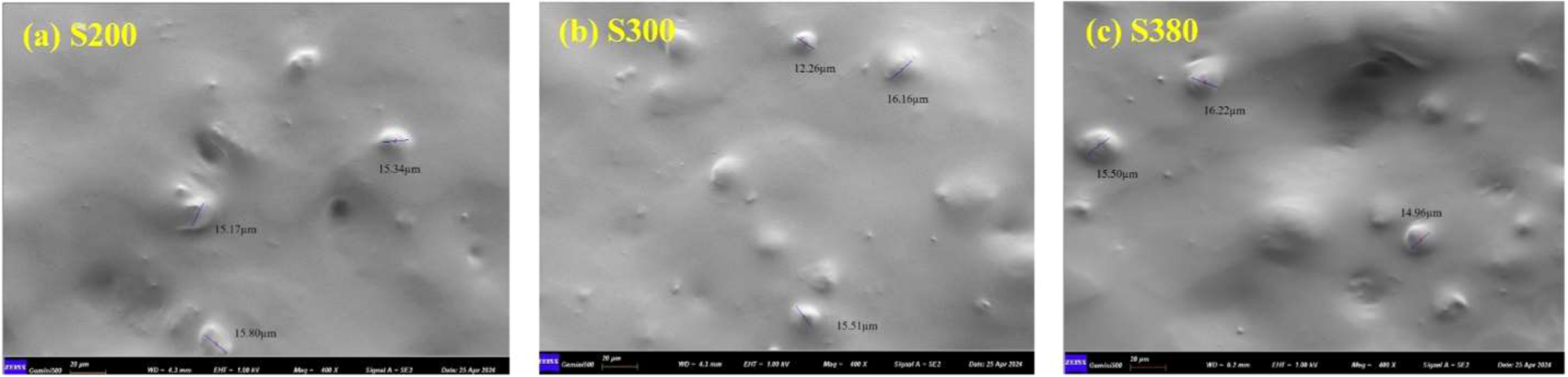
Cross-sectional morphologies of silica-filled silicone gel: samples (a) S200, (b) S300, and (c) S380.

The data listed in Table S2 indicate that *Y* is greater than *V*_filler_. Hence, based on the fitted conductivities of pure silicone gel (S000), and assuming that the conductivity of the insulating particles is much lower than that of the interface, the ion conductivity at the interface σ_int_ and the ion diffusion coefficient at silica/silicone gel interfaces *D*_int_ are found by eqs 2 and 5–8, as shown in Table S2 and Figure 6. In fumed silica-filled silicone gel, the ion comes from the impurity ions left from the synthesis and doping process.^37^ It is revealed that the ionic diffusion process in the silicone gel matrix is slow, while that at the silica/silicone gel interface is fast. The ionic diffusion coefficient of the composites lies between those of the matrix and the interface due to the mixture effect.

### Decoupling between the Ionic Diffusion Coefficient and the Segmental Relaxation Time at the Interface

After understanding the segmental relaxation time and ionic diffusion coefficient at the silica/silicone gel interface, we further discuss for the first time how the interfacial segmental dynamics influences the interfacial ion diffusion by experiments. According to the decoupling relationship (eq 12), fitting between lg τ_int_ and lg *D*_int_, as shown in Figure 8a, remains in a linear relationship, where squares of correlation coefficients are 0.999, 0.997, and 0.994 for samples S200, S300, and S380. The decoupling indexes are extracted and plotted in Figure 8b. It is found that upon increasing the specific surface area of silica, the decoupling index *n* is closer to 0, which indicates that the movement of the polymer chains at the interface gradually dominates the ion diffusion process. Furthermore, according to eq 12, when the τ_int_ is less than 1, reducing *n* increases both *D*_int_ and ion transport at the interfaces. This situation conforms to Figure 8, where τ_int_ in our case is far less than 1. This also implies that as the specific surface area of silica increases, ion diffusion at the interface slows relative to the movement of polymer chains at the interface. Therefore, there must be a factor enhancing ion transport that is independent of the segmental dynamics of the polymer chains at the interface. This effect is more pronounced in silica, with a lower specific surface area.

**Figure 8:**
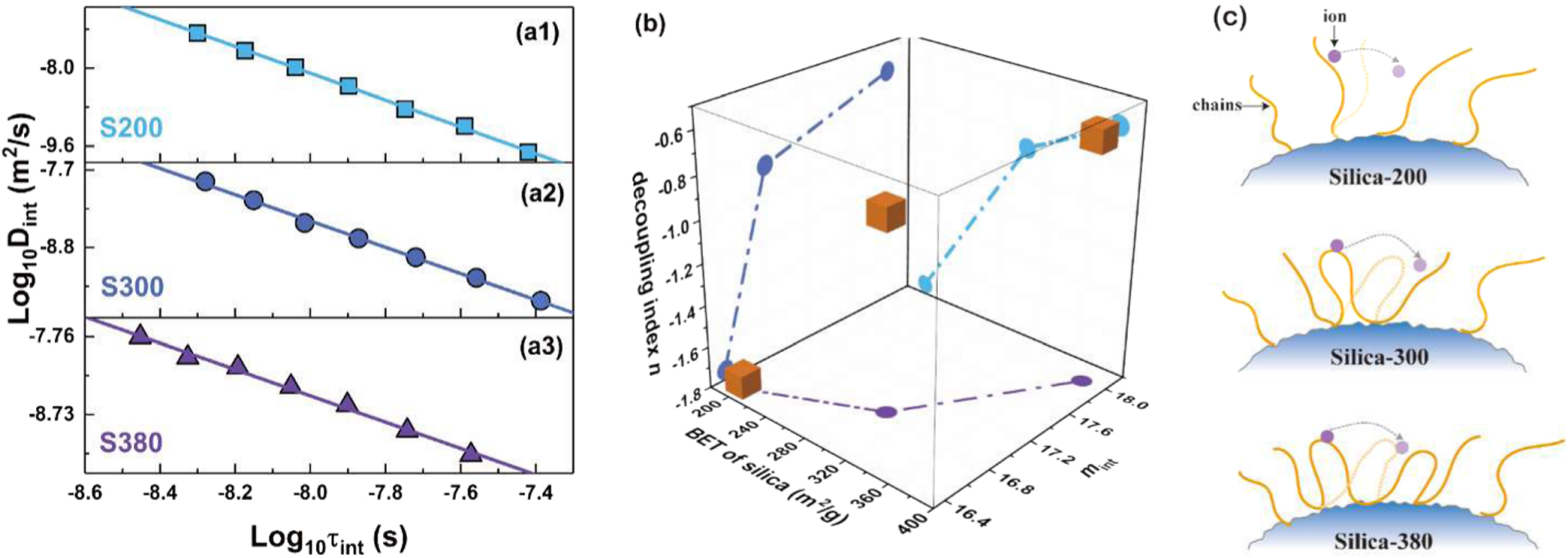
(a1–a3) Relationship between ionic diffusion coefficients and relaxation time of polymers at silica/silicone gel interfaces, where the relaxation time of α_int_ is predicted by the VTF equation. (b) Relationship among the specific surface area of silica, the cooperative rearrangement ability of chains at interfaces, and the decoupling index *n*. (c) Scheme of the decoupling relationship at the interfaces of the indicated samples.

The definition of the ionic diffusion coefficient is expressed as^6,42^

15 $$D_{\mathrm{int}}=\frac{\lambda^{2}}{6\tau_{ion}\left(T\right)}\approx \frac{\lambda^{2}}{6}\left[\frac{1}{\tau_{\mathrm{int}}\left(T\right)}+\frac{1}{\tau_{b}}\exp\left(-\frac{E_{\sigma }\left(T\right)}{k_{B}T}\right)\right]\propto \tau_{\mathrm{int}}^{n-1}\left(T\right)$$

where λ is the length of the ion jump, τ_ion_ is the time of ion jumps, τ_b_ is a time constant, and *E*_σ_ is the energy barrier for ion jumps in a frozen structure. The first and second terms represent the contribution of segmental chain relaxation through the liquidlike mechanism and ion diffusion in an essentially frozen structure through the solidlike mechanism, respectively. If *n* = 0, it indicates that the second term, ion diffusion in a frozen structure, can be neglected and the ion diffusion is completely dominated by polymer chain relaxation. If *n* is far from 0, it means that ion diffusion in a frozen structure influences the ion transport more. According to eq 15, the decoupling index depends on the contribution of the second term, ion transportation by the solidlike mechanism. Therefore, the key parameter in the second term, *E*_σ_, is further studied to reveal how the specific surface area of the particles affects the energy barrier^43^

16 $$E_{\sigma }=\frac{\beta q_{1}q_{2}}{4\pi \varepsilon_{0}\varepsilon \left(R_{1}+R_{2}\right)}+G4\pi \lambda {\left(R_{1}-R_{D}\right)}^{2}$$

The first term is the electrostatic interaction of a mobile ion with a charge *q*_1_ with a radius *R*_1_ in contact with another ion with a charge *q*_2_ and a radius *R*_2_, while β is the “Madelung” constant and *ε* is the dielectric constant around the frequency of ion rearrangements. The second term is the elastic force, where *G* is the high-frequency shear modulus and *R*_D_ is related to the free volume in polymers.

For the first term, the ranking of the dielectric constant in the DC region decreases from sample S200 to sample S300 and then to sample S380 (Table S2). This indicates that increasing the specific surface area amplifies the electrostatic interaction effect, thereby raising the energy barrier.

For the second term, as for the free volume parameter and high-frequency shear modulus, it is noted in Sokolov’s work that a higher polymer fragility index *m* corresponds to more rigid chains, resulting in looser structures, more frustrated packings, and lower packing efficiency.^44^ This provides enough space (free volume) for ions to diffuse more easily even when the segmental relaxation is very slow, increasing the decoupling degree.^12,13,45,46^ Note that the frustration of the chain packing locally decreases the shear modulus.^47^ Therefore, a greater *m* corresponds to a longer free volume and a reduced shear modulus. Since *m*_int_ in this work also represents the cooperative rearrangement ability of interfacial polymer chains, which is also relevant to chain rigidity, we try to establish the relationship between *m*_int_ in this paper and *m* in Sokolov’s work. Thus,^12^

17 $$m=\frac{\mathrm{dlog}\tau }{d\left(T_{g}/T\right)}|T=T_{g}$$

where τ is the relaxation time of segments. Experimenting τ_int_ of eq 1 in eq 17, we obtain

18 $$m=\frac{BT_{0}T_{g}}{{\left(T_{g}-T_{0}\right)}^{2}}\cdot \log e$$

Combining with eq 14,

19 $$m=\log e\cdot \frac{590T_{0}T_{g}}{\left(m_{\mathrm{int}}-16\right)\left(T_{g}-T_{0}\right)}$$

From eq 19, it can be found that the larger the *m*_int_, the lower the *m* is. Therefore, with increasing the specific surface area of silica, *m*_int_ increases and *m* decreases, which indicates that the interfacial polymer chains surrounding the silica with a high specific surface area contribute to a reduced free volume, a higher shear modulus, and the elastic force and energy barrier. It is also reflected in the relationship between *m*_int_ and the decoupling index *n* shown in Figure 8b, which is consistent with the analysis of PolyILs in ref 13.^13^

Thus, the increased specific surface area of silica raises the energy barrier *E*_σ_ and reduces the second term in eq 15, and consequently, the contribution of ion diffusion in the frozen structures decreases. The effect of segmental dynamics in the first term becomes considerable, attenuating the decoupling degree, as shown in Figure 8c. Meanwhile, with the increase of the specific surface area of silica, the improved electrostatic interaction and elastic force at interfaces impede the ion diffusion at interfaces. All of these data are consistent with the results shown in Figure 8b.

## Conclusions

In this paper, a method for calculating the ionic diffusion coefficient at a particle/polymer interface is proposed based on the effective medium theory and experimental results. Silica particles with varying specific surface areas are blended with silicone gel to create model composites, enabling an investigation of the relationship between segmental dynamics and ion diffusion at these interfaces. The increased specific surface area of silica generates more loop-type structure chains at the interface, accelerating the segmental movement at interfaces. Furthermore, the enhanced chain flexibility, combined with the low dielectric constant, strengthens electrostatic interactions and elastic forces at the interface. This increases the ion jump energy barrier, reduces the facilitation of ion diffusion, and emphasizes the influence of the interface chain movement on ion diffusion. This work provides valuable insights into how segmental dynamics affects the ion diffusion process at interfaces, paving the way for innovative strategies to control ionic conductivity in composites.

## Supplementary Material



## Notes

The definition of symbols in the article; the FTIR spectrum of silica and silicone gel; calculation about interfacial adhesion between silica and silicone gel; and the calculated detailed parameters for interfacial ion diffusion coefficients (PDF)
