# Supplementary material for: Decoupling of Interfacial Ionic Diffusion from Segmental Dynamics in Silica-Filled Silicone Gel through Experiments
Source: Langmuir. 2025 Jun 17;41(27):17875–84. doi: 10.1021/acs.langmuir.5c01650 (PMC12269070; doi:10.1021/acs.langmuir.5c01650)
Supplement: Supplementary file 1 [file la5c01650_si_001.pdf]

Supporting Information

Decoupling of interfacial ionic diffusion from segmental dynamics in silica filled silicone gel through experiments

Ying Lin[1], Chuanle Heng[1], Yuhao Liu[2], Yifan Xu[1], Tao Wen[1*], Antonio Facchetti[3*], Lijian Ding[1]

1 School of Electrical and Automation Engineering, Hefei University of Technology, Hefei, 230009, China;
2 College of Electrical Engineering and Automation, Fuzhou University, Fuzhou 350108, China
3 School of Materials Science and Engineering, Georgia Institute of Technology, Atlanta, 30332, GA, United States

Number of pages: 5

Number of figures: 2

Number of tables: 2

Table of Contents

Table of Contents	Page No.
The definition of symbols in the article	S2-S3
The FTIR spectrum of silica and silicone gel	S3
Calculation about interfacial adhesion between silica and silicone gel	S3-S4
The calculated detailed parameters for interfacial ion diffusion coefficients	S5

* Corresponding author: tao-wen@hfut.edu.cn; afacchetti6@gatech.edu

Table S1 The definition of symbols in the article

Symbol	Definition
τ_{int}	Relaxation time of relaxation α_{int}
τ_0	Constant relaxation time
T_0	Vogel temperature
B	Fragile strength
T	Absolute temperature
D_{int}	Ionic diffusion coefficient at the interface
σ_{int}	Ionic conductivity at the interface
k_B	Boltzmann's constant
q	Charge of ions
n_0	Concentration of ions
D_{cp}	Ionic diffusion coefficient of the composites
σ_{cp}	Conductivities of the composites
$(\tan\theta)_{max}$	Maximum value of dielectric loss in the frequency range of electrode polarization
f_{max}	Frequency corresponding to $(\tan\theta)_{max}$
L	Thickness of tested composites
Y	Volume fraction of particles in a composite unit
R	Radius of the particles
d	Thickness of the interface layer
V_{filler}	Volume fraction of fillers in the whole sample
m_{filler}	Weight of fillers
ρ_{filler}	Density of fillers
$\Delta\varepsilon_{matrix}$	Relaxation strength of polymer chains in matrixes
$\Delta\varepsilon_{int}$	Relaxation strength of interfacial polymer chains
p_c	Continuous percolation threshold for the fillers
σ_c^a , σ_0^a ,σ_c^b , σ_2^b	Modified conductivity parameters
σ_0	Conductivity of the matrix
σ_c	Equivalent conductivity of the composite unit
σ_{filler}	Conductivity of the fillers
P_c	Percolation threshold of a general random mixture
n	Decoupling degree between the molecular chain movement and ion diffusion process
ε^*	Complex permittivity
ε_∞	Permittivity at high frequency
s	Number of relaxation peaks
$\Delta\varepsilon_i$	Dielectric strength of *i*th relaxation
τ_i	Relaxation time of *i*th relaxation
α_i	Parameter depending on the shape of *i*th relaxation
ω	Angular frequency
ε_0	Vacuum permittivity
σ_{dc}	Dc conductivities
m_{int}	Cooperative rearrangement ability of interface polymer chains
λ	Length of the ion jump

τ_{ion}	Time of ion jumps
τ_b	Time constant
E_σ	Energy barrier for ion jumps in a frozen structure
q_1, q_2	A charge
R_1	Radius of a charge q_1
R_2	Radius of a charge q_2
β	"Madelung" constant
ε	Dielectric constant
G	High-frequency shear modulus
R_D	Related to the free volume in polymers
m	Polymer fragility index
τ	Relaxation time of segments

The FTIR spectrum of silica and silicone gel

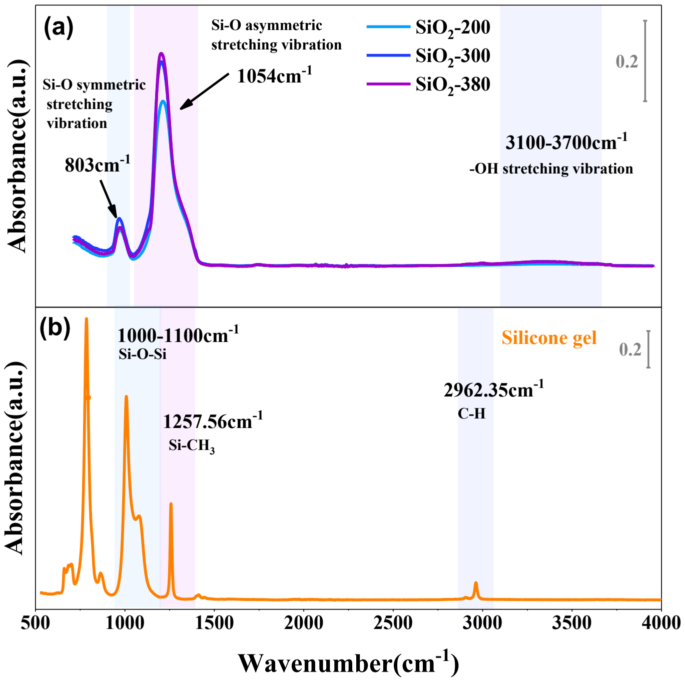

Figure S1 The FTIR spectrum of silica with different BET (a) and silicone gel (b)

Fourier transform infrared spectroscopy (FTIR) Tensor 27 spectromer (Brucker Optics, Germany) was used to observe the functional groups insides the silica and silicone gel in a spectral range from 500cm^{-1} to 4000 cm^{-1} by attenuated total reflection mode. In Figure S2 (a), it can been found Si-O bond vibration and -OH vibration in silica. In Figure S2(b), there are Si-O-Si backbonds, Si-CH_3 side chains and C-H bonds in silicone gel.

Calculation about interfacial adhesion between silica and silicone gel

To investigate the interfacial adhesion between silica and silicone gel, molecular dynamics (MD) simulations were performed. The initial molecular configurations are presented in Figure R2.1 (a) and (b). Silica was cleaved along the (001) surface and expanded into supercells, while 50 polydimethylsiloxane (PDMS) chain representing

the silicone gel backbone, were packed into amorphous cells, followed by 500 steps of energy minimization. The silica/PDMS interface model was constructed using the “Build Layer” tool with dimensions of $a = b = 30$ Å, $c = 133$ Å, and angles $\alpha = \beta = 90°$, $\gamma = 120°$ as shown in Figure R2.1(c). The interfacial structure was geometrically optimized using a smart minimization algorithm, and the configuration with the lowest energy was selected following an annealing cycle from 298 K to 500 K and back to 298 K, employing a 1.0 fs time step until energy and temperature convergence. Subsequently, MD simulations were carried out in the NVT ensemble for 2.0 ns with a time step of 1.0 fs. Temperature was controlled using a Nosé thermostat, and pressure regulation was applied via the Berendsen barostat. All simulations employed the COMPASS force field [1].

The adhesion energy is used to characterize the adhesion property of silica/polydimethylsiloxane interface according to reference [2], as shown in Figure R2.1 (d). The calculated average value is 0.149 kcal/mol/Å^2, which shows that polydimethylsiloxane is adsorbed on the surface of filler. Our discussion in Section 4.2 is verified.

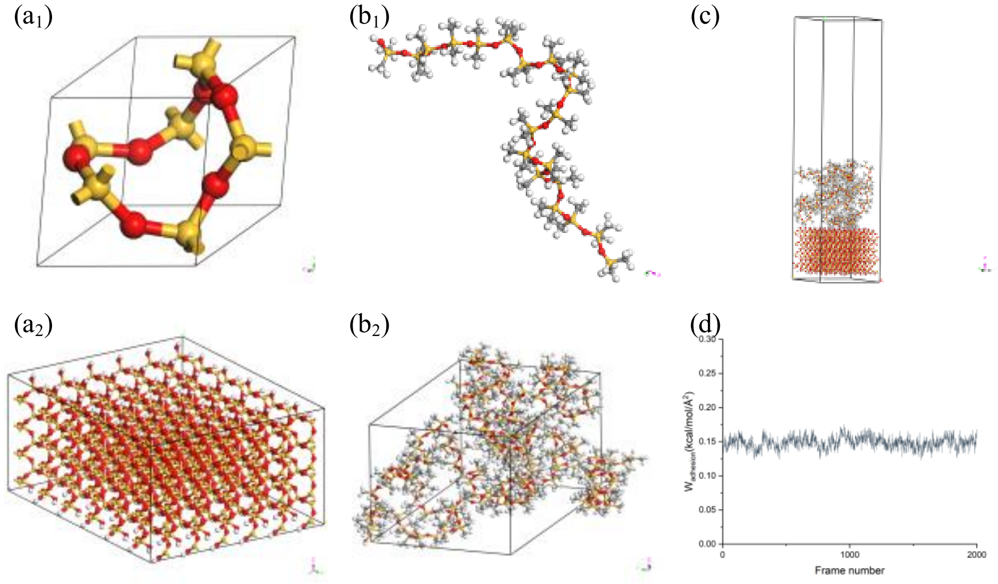

Figure S2 The initial configurations of silica (a1) and silica supercells (a2), polydimethylsiloxane (b1) and the configurations of polydimethylsiloxane amorphous cells (b2), and the silica/polydimethylsiloxane interface model (c), and adhesion energy of silica/polydimethylsiloxane interface (d).

(1) Liu, Y.; Lin, Y.; Cao, B.; Wu, K.; Wang, L. Enhancement of Polysiloxane/Epoxy Resin Compatibility through an Electrostatic and van Der Waals Potential Design Strategy. *Polym Test* **2023**, *117*, 107820. https://doi.org/10.1016/j.polymertesting.2022.107820.
(2) Xu, G.; Wang, H. Study of Cohesion and Adhesion Properties of Asphalt Concrete with Molecular Dynamics Simulation. *Comput Mater Sci* **2016**, *112*, 161–169. https://doi.org/10.1016/j.commatsci.2015.10.024.

Table S2 The calculated detailed parameters for interfacial ion diffusion coefficients, including charge concentration of ions q^2n_0, filler volume fraction V_{filler}, relaxation strength of matrix polymers $\Delta\varepsilon_{\text{matrix}}$ and interfaces $\Delta\varepsilon_{\text{int}}$, the interfacial thickness d, volume fraction of particles in a composite unit Y, the interfacial conductivity σ_{int}, static real part of permittivity $\varepsilon_{\text{static}}$

Sample	Temperature (°C)	q^2n_0(C^2/m^3)	V_{filler}	$\Delta\varepsilon_{\text{matrix}}$	$\Delta\varepsilon_{\text{int}}$	d (μm)	Y	σ_{int}(S/m)	$\varepsilon_{\text{static}}$
**S200**	50	8.44E-21	4.18E-01	4.5E-01	2.00E-02	1.49E-01	9.44E-01	3.54E-10	424.01
	60	4.17E-21	4.18E-01	4.5E-01	2.00E-02	1.49E-01	9.44E-01	5.83E-10	423.97
	70	5.44E-21	4.18E-01	5.0E-01	2.00E-02	1.35E-01	9.49E-01	1.66E-09	423.99
	80	5.6E-21	4.18E-01	6.0E-01	2.00E-02	1.14E-01	9.57E-01	4.93E-09	424.05
	90	4.4E-21	4.18E-01	6.0E-01	2.00E-02	1.14E-01	9.57E-01	9.04E-09	424.02
	100	3.59E-21	4.18E-01	6.0E-01	2.00E-02	1.14E-01	9.57E-01	1.58E-08	423.99
	110	2.54E-21	4.18E-01	6.0E-01	2.00E-02	1.14E-01	9.57E-01	2.53E-08	423.96
**S300**	50	2.19E-21	4.18E-01	3.1E-01	2.25E-02	2.23E-01	9.14E-01	1.35E-10	423.735
	60	3.21E-21	4.18E-01	3.0E-01	2.25E-02	2.30E-01	9.12E-01	4.11E-10	423.69
	70	6.32E-21	4.18E-01	3.3E-01	2.25E-02	2.11E-01	9.18E-01	1.54E-09	423.68
	80	9.9E-21	4.18E-01	3.3E-01	2.25E-02	2.11E-01	9.18E-01	4.33E-09	423.64
	90	9.39E-21	4.18E-01	3.0E-01	2.25E-02	2.30E-01	9.12E-01	6.61E-09	423.58
	100	6.33E-21	4.18E-01	3.0E-01	2.25E-02	2.30E-01	9.12E-01	9.15E-09	423.55
	110	4.86E-21	4.18E-01	3.0E-01	2.25E-02	2.30E-01	9.12E-01	1.26E-08	423.52
**S380**	50	2.05E-21	4.18E-01	1.8E-01	2.35E-02	3.96E-01	8.62E-01	2.69E-10	283.6
	60	1.49E-21	4.18E-01	1.8E-01	2.35E-02	3.96E-01	8.62E-01	3.81E-10	283.57
	70	1.78E-21	4.18E-01	1.8E-01	2.35E-02	3.96E-01	8.62E-01	9.25E-10	283.535
	80	2.11E-21	4.18E-01	1.8E-01	2.35E-02	3.96E-01	8.62E-01	1.79E-09	283.5
	90	1.78E-21	4.18E-01	1.8E-01	2.35E-02	3.96E-01	8.62E-01	2.56E-09	283.47
	100	2.07E-21	4.18E-01	1.8E-01	2.35E-02	3.96E-01	8.62E-01	3.86E-09	283.44
	110	1.39E-21	4.18E-01	1.8E-01	2.35E-02	3.96E-01	8.62E-01	4.55E-09	283.41
